# Chronic Kidney Disease Severity and 30-Day Outcomes After Total Ankle Arthroplasty: An NSQIP Study

**DOI:** 10.1177/24730114251398767

**Published:** 2025-12-19

**Authors:** George T. Liu, Cheng Zheng, Michael Huo, Jianghu James Dong

**Affiliations:** 1 Department of Orthopaedic Surgery, University of Southwestern Medical School, Dallas, TX, USA; 2 Department of Biostatistics, University of Nebraska Medical Center, Omaha, NE, USA

**Keywords:** chronic kidney disease, total ankle arthroplasty, postoperative complications, length of hospital stay, unplanned return to the operating room

## Abstract

**Background:**

Chronic kidney disease (CKD) is a recognized risk factor for adverse outcomes in total hip and knee arthroplasties; however, its impact on total ankle arthroplasty (TAA) outcomes is limited. This study investigates the association between CKD severity and 30-day postoperative outcomes following TAA.

**Methods:**

We analyzed the American College of Surgeons National Surgical Quality Improvement Program (NSQIP) database for primary TAAs between 2006 to 2021. Estimated glomerular filtration rate (eGFR) was calculated, categorizing patients into CKD stages. Univariable analysis assessed associations between eGFR categories, demographic characteristics, and outcomes. Multivariable regression models were used to identify predictors of hospital length of stay, unplanned return to the operating room, and overall complications.

**Results:**

Of the 1678 eligible primary TAA cases, 675 were G1, 806 G2, 136 G3a, 50 G3b, 7 G4, and 4 G5. Significant differences across eGFR categories were found for age, sex, race, anesthesia type, diabetes, hypertension, postoperative dialysis, American Society of Anesthesiologists class, blood urea nitrogen, creatinine, white blood cell count, and hematocrit. The overall complication rate was 3% (53/1678). Rates of myocardial infarction, unplanned return to surgery, and hospital length of stay differed significantly among eGFR groups. Multivariable negative binomial regression identified predictors of longer hospital stay including age 80-89 years, male sex, American Indian / Alaska Native race, unknown race, partial functional dependency, monitored anesthesia care/intravenous sedation, insulin-dependent diabetes, greater or equal to 2 direct complications, and 1 indirect complication. Notably, CKD stage G3b was associated with significantly longer stays compared with G1. Logistic regression revealed that 1 or greater or equal to 2 direct complications and CKD stages G4+G5 significantly predicted unplanned return to surgery. Smokers experienced higher overall complication rates.

**Conclusion:**

In this retrospective observational study, we found that CKD severity significantly impacts postoperative outcomes following TAA, with advanced stages linked to prolonged hospital stays and increased risk of unplanned return to surgery.

**Level of Evidence:**

Level III, retrospective cohort series.

## Graphical abstract

**Graphical Abstract:**
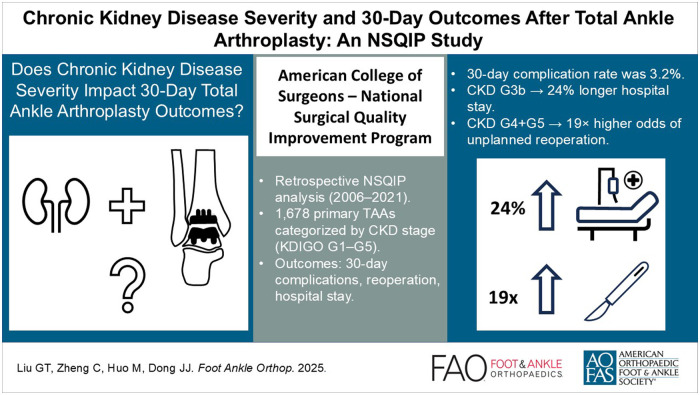

## Introduction

Between 2016 and 2018, a total of 58.5 million US adults (24% of all adults) were diagnosed with arthritis, and 25.7 million (10%) reported arthritis-attributable activity limitations.^41^ Lower extremity arthritis significantly impairs health-related quality of life (HRQoL) and ability to work compared with the general population without osteoarthritis.^1,31,35^ Although symptomatic ankle arthritis is estimated at 3% of the population, which is lower than knee (16%) and hip arthritis (9%), the negative impact on HRQoL in patients with end-stage ankle arthritis is equivocal to end-stage hip arthritis.^14,18,21,34^

Total ankle arthroplasty has emerged as an effective treatment for advanced tibiotalar arthritis. Between 2009 and 2019, a total of 41 060 primary TAAs were performed in the United States.^25^ The change in annual case volumes from 2180 to 5147 represents a 136.1% increase, likely attributed to improved implant design, patient selection, and implant survivorship.^32,44^ With the average TAA recipient being 63.6 years old, there is an associated burden of age-related comorbidities such as obesity, chronic pulmonary disease, diabetes mellitus, and CKD, which may affect surgical outcomes.^9,36^

Chronic kidney disease, characterized by the progressive loss of renal function, affects an estimated 843.6 million people worldwide and more than 37 million Americans.^7,22^ In joint replacement surgery, CKD has been associated with increased risks of postoperative complications including bleeding requiring blood transfusions, readmission, prolonged hospital stays, and genitourinary complications following total hip arthroplasty (THA) and total knee arthroplasty (TKA).^17,33,43^

The impact of CKD on joint replacement outcomes has been well documented for THA and TKA.^3,11,12,15,17,19,23,26^ A meta-analysis and systematic review have reported that patients with CKD undergoing total joint replacements have significantly higher risks of surgical site infection, need for transfusion, readmission, and mortality compared to those without CKD.^11^ Consequently, the total cost of care for arthroplasty in patients with end-stage renal disease (ESRD) requiring hemodialysis has been reported up to $19 490 USD higher compared to those without CKD or not requiring hemodialysis.^17,42^

Although the impact of CKD on THA and TKA outcomes is well documented, its influence on TAA outcomes remains poorly understood. Previous studies examining renal disease and TAA outcomes characterized renal disease as either present or absent without accounting for the severity of CKD. Therefore, the goal of this study is to evaluate the impact of CKD severity on 30-day postoperative complications in patients undergoing TAA. We specifically examined predictors of prolonged hospital length of stay, unplanned reoperation, and overall complication rate across various stages of CKD severity undergoing TAA.

## Materials and Methods

### Data Source and Patient Selection

We reviewed patient records from the American College of Surgeons NSQIP database, a validated, risk-adjusted national quality improvement repository that includes over 9.6 million cases from more than 700 participating US hospitals.^2^ Data are collected by clinically trained staff and include demographic, preoperative, intraoperative, and 30-day postoperative outcomes. Annual audits ensure data quality and abstraction reliability. Because the NSQIP database contains deidentified patient information, formal IRB approval was not required.

Patients who underwent primary TAA between January 2006 and December 2021 were identified using Current Procedural Terminology code 27702.

Renal function was estimated using the Chronic Kidney Disease–Epidemiology Collaboration (CKD-EPI) formula recommended by the 2012 Kidney Disease: Improving Global Outcomes (KDIGO) guidelines for its accuracy and lower false-positive rates.^29^

Patients were excluded if they were aged >90 years as NSQIP does not report precise age above 90 or if laboratory data necessary to calculate the eGFR were missing.

CKD was defined as an eGFR less than 60 mL/min/1.73 m^2^ persisting for at least 3 months.^10^ Patients were categorized by CKD stage using the 2012 KDIGO classification:

G1: greater or equal to 90 mL/min/1.73 m^2^ (normal or high)
G2: 60-89 mL/min/1.73 m^2^ (mildly decreased)
G3a: 45-59 mL/min/1.73 m^2^ (mild to moderately decreased)
G3b: 30-44 mL/min/1.73 m^2^ (moderately to severely decreased)
G4: 15-29 mL/min/1.73 m^2^ (severely decreased)
G5: less than 15 mL/min/1.73 m^2^ (kidney failure; treatment with dialysis)

Patients with renal insufficiency were defined as having an increase in creatinine greater than 2 mg/dL from the preoperative value without requiring dialysis within 30 days of the operation. Patients who developed postoperative dialysis requiring acute renal failure (ARF) were not categorized as having CKD. End-stage renal disease was defined as an eGFR less than 15 mL/min/1.73 m^2^. Dialysis was reported if patients had peritoneal dialysis, hemodialysis, hemofiltration, hemodiafiltration, or ultrafiltration for acute or chronic kidney failure within 2 weeks of TAA.

### Variables Collected

Demographic variables included age, gender, race, height, weight, and body mass index. Medical comorbidities captured included diabetes mellitus (stratified into insulin-requiring and noninsulin-requiring), hypertension, heart failure, disseminated cancer, immunosuppressive therapy with steroids, coagulation disorders, preoperative transfusion needs, and renal failure requiring preoperative dialysis. Smoking status (current smoking within 1 year prior to surgery) and functional status (categorized as independent, partially dependent, or totally dependent based on activities of daily living) were also recorded.

Preoperative laboratory values included serum sodium, blood urea nitrogen (BUN), creatinine, white blood cell (WBC) count, hematocrit (HCT), and platelet count. Treatment variables recorded were American Society of Anesthesiologists (ASA) classification, principal anesthesia technique, inpatient vs outpatient status, and total operative time.

### Outcome Measures

The outcomes represented the presence of a complication within the 30-day postoperative period. These outcome variables of interest included superficial surgical site infection, deep surgical site infection, wound dehiscence, deep vein thrombosis, pulmonary embolism, bleeding requiring intraoperative/postoperative transfusions, sepsis, septic shock which were categorized as direct complications related to the surgery. Pneumonia, ARF requiring postoperative dialysis, urinary tract infection (UTI), cerebral vascular accident (CVA), and myocardial infarction (MI) was categorized as indirect complications to the surgery. Overall (total) complications were the sum of direct and indirect complications. Other outcomes reported include length of total hospital stay in days and unplanned return to the operating room which included all major surgical procedures that required the patient to be taken to the operating room.

### Statistical Analysis

Exploratory analysis examined the distribution of CKD stages and complication frequencies using descriptive statistics, charts, and summary measures. Categorical variables were compared using χ^2^ or Fisher exact tests, and continuous variables were analyzed via 1-way analysis of variance with Tukey honestly significant difference post hoc tests when applicable. An alpha level <.05 was used to determine significance.

Variables with significant associations in univariable analyses were entered into multivariable regression models. Hospital length of stay and total complications were modeled using negative binomial regression, with results expressed as incidence rate ratios (IRRs) and 95% CIs. Unplanned return to the operating room was modeled using logistic regression, with odds ratios (ORs) and 95% CIs. All analyses were conducted using SAS OnDemand for Academics (SAS Institute Inc).

## Results

### Patient Cohort

**Figure 1.**
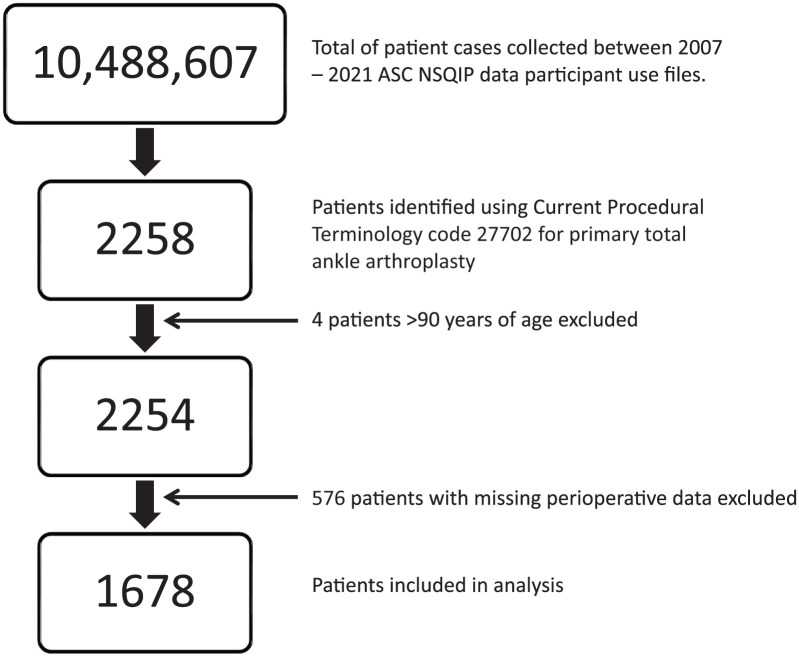
Patient selection with inclusion and exclusion criteria.

A total of 2258 patients undergoing primary TAA between January 2006 and December 2021 were initially identified. After excluding 4 patients older than 90 years and 576 cases with missing laboratory data needed for eGFR calculation, 1678 patients were included (Figure 1). The mean age was 65.1 ± 9.8 years. Mean ages by eGFR category were 60.9 ± 9.8 (G1), 67.1 ± 8.7 (G2), 71.0 ± 7.8 (G3a), 73.4 ± 7.0 (G3b), 68.9 ± 7.4 (G4), and 62.8 ± 13.2 (G5) years. Overall, 55% of patients (918/1678) were male. Approximately 76% of the patients (1281/1678) were White, 3.5% (58/1678) were Hispanic, 2.8% (47/1678) were African American, 2% (27/1678) were Native Hawaiian/Pacific Islander/Asian, 0.1% (2/1678) were American Indian or Alaska Native, and 16% (261/1678) were unknown/unreported. Inpatient procedures comprised 79% (1326/1678) of cases. All 4 patients in the G5 group were on dialysis and inpatient. Significant differences across eGFR categories were observed in age, sex, race, anesthesia type, diabetes, hypertension, postoperative dialysis, ASA class, BUN, creatinine, WBC count, and HCT (Table 1).

**Table 1.** Demographics of Patients Who Underwent Total Ankle Arthroplasty.^a^

Variable	G1 (≥90 mL/min)	G2 (60-89 mL/min)	G3a (45-59 mL/min)	G3b (30-44 mL/min)	G4 (15-29 mL/min)	G5 (<15 mL/min)	*P* Value
Subjects, n	675	806	136	50	7	4	
Age, y	60.9 ± 9.79	67.1 ± 8.73	71.0 ± 7.83	73.4 ± 7.01	68.9 ± 7.40	62.8 ± 13.15	**.001** ^b^
Age category							
≤50 y	93 (13.78)	27 (3.35)	1 (0.74)	0	0	1 (25.00)	**.001** ^b^
51-60 y	208 (30.81)	148 (18.36)	13 (9.56)	2 (4.00)	1 (14.29)	0	
61-70 y	265 (39.26)	344 (42.68)	48 (35.29)	17 (34.00)	2 (28.57)	2 (50.00)	
71-80 y	106 (15.70)	245 (30.40)	60 (44.12)	21 (42.00)	4 (57.14)	1 (25.00)	
80-89 y	3 (0.44)	42 (5.21)	14 (10.29)	10 (20.00)	0	0	
Male	384 (56.8)	444 (55.1)	67 (49.3)	16 (32.0)	4 (57.1)	2 (50.0)	**.014** ^b^
BMI	31.3 ± 5.86	31.1 ± 5.92	32.0 ± 5.73	32.4 ± 6.80	31.1 ± 6.43	33.1 ± 7.52	.418^b^
Race							
White	511 (75.7)	610 (75.7)	116 (85.3)	39 (78.0)	5(71.4)	1 (25.0)	**.001** ^b^
Hispanic	33 (4.9)	25 (3.1)	0	0	0	0	
Black	19 (2.8)	18 (2.2)	3 (2.2)	3 (6.0)	1 (14.3)	3 (75.0)	
NHPI Asian	17 (2.5)	10 (1.2)	0	0	0	0	
AI or AN	2 (0.3)	0	0	0	0	0	
Unknown	93 (13.8)	143 (17.7)	17 (12.5)	8 (16.0)	1 (14.3)	0	
Anesthesia							
General	590 (87.4)	702 (87.1)	116 (85.3)	36 (72.0)	7 (100)	4 (100)	**.017** ^b^
Spinal	49 (7.3)	44 (5.5)	10 (7.4)	9 (18.0)	0	0	
MAC/IV	16 (2.4)	42 (5.2)	5 (3.7)	3 (6.0)	0	0	
Region	18 (2.7)	17 (2.1)	4 (2.9)	1 (2.0)	0	0	
Epidural	1 (0.2)	1 (0.1)	1 (0.7)	0	0	0	
Other/unknown	1 (0.15)	0	0	1 (2.0)	0	0	
Hospital status							
Inpatient	521 (77.19)	641 (79.53)	108 (79.41)	46 (92.00)	6 (85.71)	4 (100)	.155^b^
Outpatient	154 (22.81)	165 (20.47)	28 (20.59)	4 (8.00)	1 (14.29)	0	
Diabetes							
None	591 (87.6)	717 (89)	111 (81.6)	32 (64.0)	6 (85.7)	2 (50)	**.001** ^b^
Non-insulin	72 (10.7)	70 (8.7)	16 (11.8)	9 (18.0)	1 (14.3)	0	
Insulin	12 (1.8)	19 (2.4)	9 (6.6)	9 (18.0)	0	2 (50)	
Smoker							
Yes	68 (10.1)	47 (5.8)	8 (5.9)	1 (2.0)	1 (14.3)	1 (25)	.008^c^
No	607 (89.9)	759 (94.2)	128 (94.2)	49 (98.0)	6 (85.7)	3 (75)	
Functional status							
Independent	666 (98.7)	789 (97.9)	133 (97.8)	47 (94.0)	7 (100)	4 (100)	.13^c^
Partial Dependent	4 (0.6)	9 (1.1)	3 (2.2)	3 (6.0)	0	0	
Unknown	5 (0.7)	8 (1.0)	0	0	0	0	
CHF							
Yes	1 (0.2)	2 (0.3)	1 (1.5)	1 (2.0)	0	0	.065^c^
No	674 (99.9)	804 (99.8)	134 (98.5)	49 (98.0)	7 (100)	4 (100)	
HTN							
Yes	337 (49.9)	489 (60.7)	102 (75.0)	48 (96.0)	6 (85.7)	4 (!00)	**.001** ^c^
No	338 (50.1)	317 (39.3)	34 (25.0)	2 (4.0)	1 (14.3)	0	
Dialysis							
Yes	0	0	0	0	0	3 (75.0)	**.001** ^c^
No	675 (100)	806 (100)	136 (100)	50 (100)	7 (100)	1 (25.0)	
Disseminated cancer							
Yes	2 (0.3)	1	0	0	0	0	.721^c^
No	673 (99.7)	805 (99.9)	136 (100)	50 (100)	7 (100)	4 (100)	
Steroids							
Yes	37 (5.5)	41 (5.1)	9 (6.6)	4 (8.0)	1 (14.3)	0	.545^c^
No	638 (94.5)	765 (94.9)	127 (93.4)	46 (92.0)	6 (85.7)	4 (100)	
Bleeding disorder							
Yes	15 (2.2)	25 (3.1)	1 (0.74)	3 (6.0)	0	0	.286^c^
No	660 (97.8)	781 (96.9)	135 (99.3)	47 (94.0)	7 (100)	4 (100)	
ASA							
1	26 (3.9)	27 (3.4)	2 (1.5)	0	0	0	**.001** ^b^
2	398 (59.0)	431 (53.5)	51 (37.5)	13 (26.0)	1 (14.3)	0	
3	245 (36.3)	337 (41.8)	81 (59.6)	35 (70)	6 (85.7)	2 (50)	
4	6 (0.9)	11 (1.4)	2 (1.5)	2 (4)	0	2 (50)	
Sodium	139.6 ± 2.56	139.6 ± 2.86	139 ± 2.93	139.7 ± 2.69	140 ± 2.24	139.3 ± 5.32	.993^b^
BUN	15.6 ± 4.57	18.3 ± 5.46	23.1 ± 7.28	32.3 ± 10.38	37.4 ± 8.62	37.8 ± 24.17	**.001** ^b^
Creatinine	0.76 ± 0.12	0.97 ± 0.15	1.26 ± 0.21	1.51 ± 0.21	2.37 ± 0.15	5.43 ± 1.71	**.001** ^b^
WBC count	6.99 ± 2.44	6.80 ± 1.87	7.53 ± 2.11	6.67 ± 1.75	7.67 ± 1.94	6.75 ± 0.53	**.017** ^b^
HCT	41.9 ± 3.86	42.2 ± 3.96	40.8 ± 4.19	38.8 ± 4.03	37.9 ± 2.64	33.2 ± 2.30	**.001** ^b^
Platelets	246.6 ± 73.14	238.9 ± 62.72	240.6 ± 69.78	224.3 ± 62.83	236.7 ± 55.62	230.3 ± 73.08	.168^b^
eGFR (CKD-EPI)^d^	99.1 ± 6.70	76.4 ± 8.27	53.5 ± 4.26	40.2 ± 3.45	25.7 ± 2.43	10.5 ± 3.74	**.001** ^b^

Abbreviations: AI, American Indian; AN, Alaska Native; ASA, American Society of Anesthesiologists; BMI, body mass index; BUN, blood urea nitrogen; CHF, Congestive heart failure; eGFR, estimated glomerular filtration rate; HCT, hematocrit; HTN, hypertension; MAC/IV, monitored anesthesia care/intravenous; NHPI, Native Hawaiian, Pacific Islander; WBC, white blood cell.

a Age, BMI, sodium, BUN, creatinine, WBC, HCT, platelets, and eGFR are all means with SD. The rest are n (column %). Bold *P* values indicate significance (*P* < .05).

b *P* values are from χ^2^ test.

c *P* values are from Fisher exact test.

d Estimated using the Chronic Kidney Disease–Epidemiology Collaboration (CKD-EPI) formula.

### Complications and Length of Stay

A total of 53 complications out of 1678 cases (3%) were recorded, including 40 direct and 13 indirect complications (Table 2). Direct complications comprised 19 superficial surgical site infection, 1 deep surgical site infection, 5 wound dehiscence, 5 deep vein thrombosis, 4 pulmonary embolism, 4 transfusion, 1 sepsis, and 1 septic shock. Indirect complications comprised 3 pneumonia, 7 urinary tract infection, 1 cerebral vascular accident, and 2 myocardial infarctions. Only myocardial infarction was significantly different between eGFR categories (*P* = .049). No patients required postoperative dialysis for ARF.

**Table 2.** Thirty-Day Complications and Outcomes of Patients Who Underwent Total Ankle Arthroplasty.^a^

Variable	G1 (≥90 mL/min)	G2 (60-89 mL/min)	G3a (45-59 mL/min)	G3b (30-44 mL/min)	G4 (15-29 mL/min)	G5 (<15 mL/min)	*P* Value
Subjects, n	675	806	136	50	7	4	
Superficial infection							
Yes	5 (0.7)	11 (1.4)	2 (1.5)	1 (2.0)	0	0	.459^b^
No	670 (99.3)	795 (98.6)	134 (98.5)	49 (98.0)	7 (100)	4 (100)	
Deep infection							
Yes	1 (0.2)	0	0	0	0	0	.452^b^
No	674 (99.9)	806 (100)	136 (100)	50 (100)	7 (100)	4 (100)	
Dehiscence							
Yes	2 (0.3)	1 (0.1)	2 (1.5)	0	0	0	.143^b^
No	673 (99.7)	805 (99.9)	134 (98.6)	50 (100)	7 (100)	4 (100)	
DVT							
Yes	3 (0.4)	1 (0.1)	1 (0.7)	0	0	0	.337^b^
No	672 (99.6)	805 (99.9)	135 (99.3)	50 (100)	7 (100)	4 (100)	
PE							
Yes	2 (0.3)	1 (0.1)	1 (0.7)	0	0	0	.382^b^
No	673 (99.7)	805 (99.9)	135 (99.5)	50 (100)	7 (100)	4 (100)	
Blood transfusion							
Yes	1 (0.2)	1 (0.1)	2 (1.5)	0	0	0	.109^b^
No	674 (99.9)	805 (99.9)	134 (98.9)	50 (100)	7 (100)	4 (100)	
Sepsis							
Yes	1 (0.2)	0	0	0	0	0	.520^b^
No	674 (99.9)	806 (100)	136 (100)	50 (100)	7 (100)	4 (100)	
Septic shock							
Yes	1 (0.2)	0	0	0	0	0	.520^b^
No	674 (99.9)	806 (100)	136 (100)	50 (100)	7 (100)	4 (100)	
Pneumonia							
Yes	1 (0.2)	2 (0.3)	0	0	0	0	>.999^b^
No	674 (99.9)	804 (99.8)	136 (100)	50 (100)	7 (100)	4 (100)	
Postoperative dialysis/ARF							
Yes	0	0	0	0	0	0	
No	675 (100)	806 (100)	136 (100)	50 (100)	7 (100)	4 (100)	
UTI							
Yes	4 (0.6)	2 (0.3)	1 (07)	0	0	0	.535^b^
No	671 (99.4)	804 (99.8)	135 (99.3)	50 (100)	7 (100)	4 (100)	
CVA							
Yes	1 (0.2)	0	0	0	0	0	.520^b^
No	674 (99.9)	806 (100)	136 (100)	50 (100)	7 (100)	4 (100)	
MI							
Yes	1 (0.2)	0	0	1 (2.0)	0	0	**.049** ^b^
No	674 (99.9)	806 (100)	136 (100)	49 (98.0)	7 (100)	4 (100)	
Return to operating room							
Yes	2 (0.3)	9 (1.1)	1 (0.7)	0	1 (14.3)	0	**.048** ^b^
No	673 (99.7)	797 (98.9)	135 (99.3)	50 (100)	6 (85.7)	4 (100)	
Operative time, min	156.4 ± 59.43	155.0 ± 56.69	147.7 ± 50.36	143.3 ± 58.72	172.3 ± 63.19	167.3 ± 60.27	.363^c^
Hospital length of stay, d	1.6 ± 1.19	1.7 ± 1.27	1.9 ± 1.57	2.7 ± 2.03	2.3 ± 0.95	3.0 ± 2.16	**.001** ^c^
Direct complication counts							
0	663 (98.2)	792 (98.6)	129 (94.9)	49 (98.0)	7 (100)	4 (100)	.273^b^
1	9 (1.3)	13 (1.6)	6 (4.4)	1 (2.0)	0 (0.0)	0 (0.0)	
2	2 (0.3)	1 (0.1)	1 (0.7)	0 (0.0)	0 (0.0)	0 (0.0)	
3	1 (0.2)	0 (0.0)	0 (0.0)	0 (0.0)	0 (0.0)	0 (0.0)	
Indirect complication counts							
0	668 (99.0)	802 (99.5)	135 (99.3)	49 (98.0)	7 (100)	4 (100)	.330^b^
1	7 (1.0)	4 (0.5)	1 (0.7)	1 (2.0)	0 (0.0)	0 (0.0)	
Total complication counts							
0	657 (97.3)	788 (97.8)	128 (94.1)	48 (96.0)	7 (100)	4 (100)	.331^c^
1	15 (2.2)	17 (2.1)	7 (5.2)	2 (4.0)	0 (0.0)	0 (0.0)	
2	2 (0.3)	1 (0.1)	1 (0.7)	0 (0.0)	0 (0.0)	0 (0.0)	
4	1 (0.2)	0 (0.0)	0 (0.0)	0 (0.0)	0 (0.0)	0 (0.0)	
Direct complications	16 (69.6)	15 (78.9)	8 (88.9)	1 (50)	0 (0.0)	0 (0.0)	.273^c^
Indirect complications	7 (30.4)	4 (21.1)	1 (11.1)	1 (50)	0 (0.0)	0 (0.0)	.330^c^
Total complications	23 (100)	19 (100)	9 (100)	2 (100)	0 (0.0)	0 (0.0)	.331^c^

Abbreviations: ARF, acute renal failure; CVA, cerebral vascular accident; DVT, deep vein thrombosis; MI, myocardial infarction; PE, pulmonary embolism; UTI, urinary tract infection.

a Operative time (min) and hospital length of stay (days) are both means with SD. The rest are n (column %). Bold *P* values indicate significance (*P* < .05).

b *P* values are from Fisher exact test.

c *P* values are from χ^2^ test.

Thirteen patients had an unplanned return to the operating room within 30 days, which was significantly different between eGFR categories (*P* = .048). The mean operative time was 154.8 ± 57.4 minutes. Mean hospital length of stay was 1.7 ± 1.3 days, which was significantly different between eGFR categories (*P* = .001). No significant differences were seen between eGFR categories with direct, indirect, or total complications. There were 2 patient deaths but neither related to complications of their TAA.

### Multivariable Regression Analysis

#### Hospital length of stay

Negative binomial regression identified independent predictors for longer hospital stays (Table 3) including patients age 80-89 years, males, American Indian / Alaska Native patients or unknown/unreported race, partially dependent status, insulin-requiring diabetes, patients with 2 direct or 1 indirect complication, and those with CKD stage G3b were all associated with significantly higher rates of prolonged hospital stay compared to their reference categories. Patients with stage G3b CKD experienced 24% longer hospital stay compared with stage G1.

**Table 3.** Multivariable Negative Binomial Regression Assessing Risk Factors Associated With Total Length of Hospital Stay.

Parameter	IRR	95% CI		*P* Value^a^
Age				
≤50 y	1.00			
51-60 y	0.94	0.79	1.12	.489
61-70 y	0.93	0.79	1.10	.400
71-80 y	1.03	0.87	1.23	.723
80-89 y	1.47	1.17	1.84	**.001**
Sex				
Female	1.00			
Male	0.87	0.80	0.95	**.002**
Race				
White	1.00			
Hispanic	1.08	0.88	1.34	.463
Black or African American	1.05	0.82	1.35	.697
Native Hawaiian, Pacific Islander, Asian	0.93	0.67	1.30	.687
American Indian or Alaska Native	2.32	1.15	4.69	**.019**
Unknown / not reported	1.38	1.25	1.52	**<.001**
Functional status				
Independent	1.00			
Partially dependent	1.40	1.05	1.86	**.022**
Unknown dependent status	0.77	0.44	1.36	.371
Anesthesia type				
General anesthesia	1.00			
MAC/IV sedation	0.79	0.64	0.97	**.024**
Epidural	1.24	0.51	3.00	.641
Regional	0.79	0.61	1.04	.090
Spinal	1.04	0.90	1.20	.576
Other/unknown	0.86	0.32	2.32	.765
No diabetes	1.00			
Diabetes insulin	1.23	0.99	1.52	**.056**
Diabetes non-insulin	1.10	0.97	1.25	.125
Nonsmoker	1.00			
Smoke	0.94	0.81	1.10	.450
No hypertension	1.00			
Hypertension	1.08	0.99	1.17	.100
No preoperative dialysis	1.00			
Preoperative dialysis	2.02	0.41	10.03	.391
ASA class				
ASA 1	1.00			
ASA 2	1.16	0.91	1.47	.238
ASA 3	1.23	0.96	1.57	.106
ASA 4	1.18	0.79	1.75	.418
0 direct complications	1.00			
1 direct complication	1.15	0.88	1.49	.313
≥2 direct complications	1.96	1.22	3.16	**.006**
0 indirect complications	1.00			
1 indirect complication	1.54	1.04	2.27	**.030**
Preoperative WBC count	1.01	0.99	1.03	.198
Preoperative HCT	0.99	0.98	1.00	**.033**
CKD stage				
G1 stage	1.00			
G2 stage	0.99	0.90	1.08	.792
G3a stage	1.05	0.90	1.23	.529
G3b stage	1.24	1.00	1.52	**.045**
G4 stage	1.33	0.80	2.23	.275
G5 stage	0.91	0.22	3.79	.892

Abbreviations: ASA, American Society of Anesthesiologists; CKD, chronic kidney disease; HCT, hematocrit; IRR, incidence rate ratio; MAC/IV, monitored anesthesia care/intravenous; WBC, white blood cell.

a Bold *P* values indicate significance (*P* < .05).

#### Unplanned return to the operating room

Both univariable and multivariable logistic regression were performed with CKD groups G1+G2, G3a+G3b, and G4+G5 and direct complications grouped as none, 1, or greater than or equal to 2 (Table 4). Patients with 1 direct complication had 25.41 times higher adjusted odds and those with greater or equal to 2 direct complications had 54.01 times higher adjusted odds of an unplanned return to the operating room compared to subjects without direct complications both statistically significant. Patients with CKD stages G4+G5 were associated with 19.19 times higher adjusted odds of unplanned return to the operating room compared with G1+G2, which was statistically significant. Among the 13 patients who experienced an unplanned return to the operating room, reasons for reoperation were available for 11 cases. Seven were related and 4 were unrelated to the index TAA procedure and 2 were not reported (Table 5).

**Table 4.** Univariable and Multivariable Logistic Regression Assessing Risk Factors Associated With Unplanned Return to Surgery.

Parameter	Univariable OR (95% CI)	Univariable *P* Value ^a^	Multivariable OR (95% CI)	Multivariable *P* Value ^a^
Complications				
0 direct complications	1.00		1.00	
1 direct complication	20.96 (5.36-81.91)	**.001**	25.41(6.27-103.00)	**.001**
≥2 direct complications	45.42 (4.61-447.20)	**.001**	54.01 (5.34-546.76)	**.008**
CKD stages				
G1 and G2 stages	1.00		1.00	
G3a and G3b stages	0.72 (0.09-5.63)	.756	0.49 (0.06-4.06)	.508
G4 and G5 stages	13.36 (1.57-113.52)	**.018**	19.18 (2.18-168.51)	**.008**

Abbreviations: CKD, chronic kidney disease; OR, odds ratio.

a Bold *P* values indicate significance (*P* < .05).

**Table 5.** Unplanned Return to the Operating Room Within 30 Days of Total Ankle Arthroplasty.

Reasons for Reoperation	n (%)
Related to TAA	7 (54)
Unrelated to TAA (nonorthopaedic causes)	4 (31)
Reason not recorded	2 (15)

Abbreviation: TAA, total ankle arthroplasty.

#### Overall complications

Negative binomial regression (Table 6) revealed that older age and males trended toward higher overall complication rates but only smoking significantly increased the IRR of overall complications by 200.7% compared with nonsmokers (IRR = 3.01, 95% CI 1.22-7.43; *P* = .017). Patients with CKD stages G3a+G3b had a 1.9-fold higher incidence rate of overall complications compared with G1 but did not reach statistical significance. No direct or indirect complications were observed in patients with CKD stages G4+G5.

**Table 6.** Multivariable Negative Binomial Regression Assessing Risk Factors Associated With Overall Complication.

Parameter	IRR	95% CI		*P* Value
Age				
≤50 y	1.00			
51-60 y	1.94	0.38	9.80	.422
61-70 y	1.29	0.26	6.38	.751
71-80 y	2.50	0.50	12.52	.264
80-89 y	2.20	0.28	17.04	.449
Sex				
Female	1.00			
Male	1.46	0.77	2.78	.244
No diabetes	1.00			
Diabetes insulin	1.31	0.30	5.77	.725
Diabetes non-insulin	1.42	0.56	3.61	.467
Nonsmoker	1.00			
Smoker	3.01	1.22	7.43	.017
ASA class				
ASA 1	1.00			
ASA 2	1.24	0.15	10.58	.841
ASA 3	1.24	0.14	10.80	.846
ASA 4	3.47	0.21	57.06	.384
Inpatient	1.00			
Outpatient	0.78	0.34	1.80	.567
CKD stage				
G1+G2 stage	1.00			
G3a+G3b stage	1.94	0.83	4.55	.128

Abbreviations: ASA, American Society of Anesthesiologists; CKD, chronic kidney disease; IRR, incidence rate ratio.

## Discussion

CKD is a global burden that affects morbidity, mortality, health care costs, and HRQoL. In our national NSQIP cohort of 1678 primary TAAs, CKD severity was not associated with higher rates of direct or indirect complications overall; however, stage G3b independently predicted longer hospital stay and stages G4+G5 independently predicted unplanned return to the operating room. A systematic review reported 697.5 million cases of CKD globally in 2017 representing a 9% prevalence.^20^ In the United States, CKD prevalence in adults increased from 10.0% in 1988-1994 to 14% in 2017-2020.^8,13^ The severity of CKD is associated with comorbidities including cardiovascular disease, hypertension, anemia, and disorders of bone mineral metabolism that increase risk for postoperative complications and subsequent economic burden to the health care system.^4,40^

Although the impact of different stages of CKD on THA and TKA outcomes has been well documented, little has been reported on the impact of CKD severity on TAA outcomes.^3,11,12,15,17,19,24,26,40^ In our analysis of the NSQIP database, we found no significant association between CKD severity and direct or indirect complications following TAA surgery. We also did not find any association between CKD severity and indirect complications of pneumonia, urinary tract infection, cerebral vascular accident, or myocardial infarction following TAA surgery. An analysis of 2157 TAA from the Electronic Data Interchange Medicare claims data reported no significant increase in TAA failures in subjects with renal failure.^28^ In their study, renal failure was defined by the presence of ARF, CKD, or unspecified kidney failure; therefore, the individual effect of each of these subgroups of renal failure on TAA outcomes could not be determined. Sambandam et al^38^ analyzed outcomes of 5087 patients who underwent TAA from the National Inpatient Sample Database from 2016 to 2019 of which 236 of 5087 patients (5%) had CKD. Additionally, they reported that patients with CKD demonstrated 9 times higher odds of ARF and 10 times higher odds of requiring a blood transfusion when compared to patients without CKD undergoing TAA surgery. The odds ratios were unadjusted and only subgroup analysis was performed. Because CKD was not stratified into stages in their study, the impact of CKD severity on these complication outcomes could not be elucidated. Ko et al^27^ in their retrospective analysis of 456 patients who underwent TAA, of which 26 patients had CKD, identified no significant association between CKD and revision or complication rates. Criteria for diagnosis and operations definition of CKD were not included in their report. Del Balso et al^16^ reported primary and revision TAA outcomes from the 2011 to 2018 NSQIP database and found in a multivariate analysis that a more than 2-day length of hospital stay, history of smoking, bleeding disorders, hypertension, and diabetes mellitus were predictive of 30-day complications. Their series included 4 dialysis dependent patients and 1 who developed ARF postoperatively. The severity of CKD was not evaluated in their study. With the multivariable regression analysis evaluating predictors for overall complication following primary TAA, we identified that only smoking status significantly increased the incidence rate of overall complication compared to nonsmokers by 200.7%. Analyzing CKD severity, subjects with G3a+G3b stage had 1.9 times higher rate of overall complications compared with G1+G2 stage; however, it was not statistically significant. There were no occurrences of direct or indirect complications with the G4+G5 stage.

Hospital length of stay has been shown to be influenced by CKD. Sambandam et al^38^ reported that the hospital length of stay following TAA was 1.99 days for patients with CKD compared with 1.73 days for patients without CKD. In our study, the overall average length of hospital stay was 1.69 days, with a trend for increased hospital length of stay with higher CKD stages. Patients with CKD stage G3b had a 24% longer hospital stay than those with G1 having normal or mildly decreased renal function, and this was statistically significant.

Unplanned return to the operating room is a widely used quality metric of hospital-based surgical care because of its association with an identifiable discrete event linked to hospital length of stay, morbidity, mortality, and cost.^5,30,37,39^ Although the overall reoperation rate in our study was low (13/1678), patients with any direct complication or advanced CKD had a higher odds of reoperation. Specifically, patients in the G4+G5 CKD group had a 19-fold increased adjusted odds for unplanned return to the operating room compared to the G1+G2 group, which was statistically significant.

Previous investigations have reported mixed results regarding CKD and TAA outcomes. For instance, Ko et al^27^ found no significant association between CKD and revision or complication rates in a retrospective analysis of 456 TAA patients, whereas Sambandam et al^38^ reported increased odds for ARF, blood transfusion, and periprosthetic fracture in CKD patients. Unlike prior TAA studies that classified renal disease using binary categories (eg, renal failure or CKD yes/no), we calculated eGFR using the CKD-EPI equation and applied KDIGO G1-G5 staging. This approach allowed us to assess the potential dose-response relationship between CKD severity and 30-day postoperative outcomes following TAA surgery. We are the first to report that in patients undergoing TAA surgery, only specific CKD stages (G3b and G4+G5) are significantly associated with prolonged hospital stays and unplanned return to the OR, whereas the overall complication rate remains low.

## Limitations

This study has several limitations associated with large national databases. First, NSQIP does not capture details such as surgeon experience, specific postoperative management protocols, or socioeconomic factors, all of which may influence outcomes. Second, the number of TAAs in NSQIP is relatively small compared with THA and TKA, potentially limiting statistical power for subgroup analyses.^6^ Third, 576 patients were excluded because of missing laboratory data required for eGFR calculation, which may influence the outcome and significance of our univariable and multivariable regression analyses. Additionally, NSQIP provides single preoperative laboratory values; chronicity of reduced eGFR (≥3 months) could not be verified, so CKD “stages” in this study reflect eGFR categories at a single time point rather than confirmed chronic disease. Fourth, because NSQIP discontinued collecting reasons for reoperations after 2012, we were only able to identify reason for reoperation in 11 of 13 cases. Therefore, we reported reasons for unplanned return to the operating room descriptively and excluded from the statistical analysis. Fifth, the overall complication rate was low at 3.2% (53/1678), which limited the ability to derive definitive conclusions from the complication analyses. The low occurrence of individual complication events reduces statistical power resulting in wider confidence intervals and less stable estimates; therefore, these findings should be interpreted with consideration of these limitations. Lastly, although no direct or indirect complications were reported among subjects with CKD stages G4 and G5, unplanned return to the operating room in this group resulted from causes not captured by the complication variables evaluated in this study. Because NSQIP collects reoperation as an outcome separate from predefined complication fields, reoperations may occur without a corresponding “complication” entry. This represents an inherent limitation of the NSQIP database.

## Conclusions

To our knowledge, this is the first study to evaluate the influence of CKD severity on 30-day outcomes following primary TAA, using a national data set spanning 16 years. In this retrospective observational study, we found that CKD stage G3b was independently associated with longer hospital stay, and stages G4+G5 were independently associated with unplanned return to the operating room. These findings support incorporating CKD stage into preoperative risk assessment and perioperative planning for individuals undergoing TAA. Larger data sets and linkage to longer-term outcomes are needed to refine risk stratification and management.

## Supplemental Material

sj-pdf-1-fao-10.1177_24730114251398767 – Supplemental material for Chronic Kidney Disease Severity and 30-Day Outcomes After Total Ankle Arthroplasty: An NSQIP Study Supplemental material, sj-pdf-1-fao-10.1177_24730114251398767 for Chronic Kidney Disease Severity and 30-Day Outcomes After Total Ankle Arthroplasty: An NSQIP Study by George T. Liu, Cheng Zheng, Michael Huo and Jianghu James Dong in Foot & Ankle Orthopaedics

## References

[1] Alyousef YS Johnston V Smith MD. Work-related outcomes in individuals with and without lower limb osteoarthritis: an online survey. BMC Public Health. 2023;23(1):1885. doi:10.1186/s12889-023-16723-3 37773119 PMC10540324

[2] American College of Surgeons National Surgical Quality Improvement Program. User guide for the 2021 ACS NSQIP participant use data file. 2022. https://www.facs.org/media/wd2hlqzv/nsqip_puf_userguide_2021.pdf

[3] Antoniak DT Benes BJ Hartman CW Vokoun CW Samson KK Shiffermiller JF. Impact of chronic kidney disease in older adults undergoing hip or knee arthroplasty: a large database study. J Arthroplasty. 2020;35(5):1214-1221.e5. doi:10.1016/j.arth.2019.12.040 31948811

[4] Bello AK Alrukhaimi M Ashuntantang GE , et al. Complications of chronic kidney disease: current state, knowledge gaps, and strategy for action. Kidney Int Suppl (2011). 2017;7(2):122-129. doi:10.1016/j.kisu.2017.07.007 30675426 PMC6341007

[5] Birkmeyer JD Hamby LS Birkmeyer CM Decker MV Karon NM Dow RW. Is unplanned return to the operating room a useful quality indicator in general surgery? Arch Surg. 2001;136(4):405-411. doi:10.1001/archsurg.136.4.405 11296110

[6] Bohl DD Idarraga AJP Hamid KS Holmes GB Lin J Lee S. Total ankle arthroplasty is safer than total hip and knee arthroplasty in the early postoperative period. J Am Acad Orthop Surg. 2020;28(16):671-677. doi:10.5435/JAAOS-D-19-00205 32769722

[7] Centers for Disease Control and Prevention. Chronic kidney disease in the United States, 2023. US Department of Health and Human Services, Centers for Disease Control and Prevention; 2023.

[8] Centers for Disease Control and Prevention. Kidney disease surveillance system. US Department of Health and Human Services; 2024.

[9] Chan JJ Guzman JZ Garden E , et al. Economic impact of comorbidities in total ankle arthroplasty and ankle arthrodesis. Orthop Traumatol Surg Res. 2022;108(7):103133. doi:10.1016/j.otsr.2021.103133 34706289

[10] Chapter 1: Definition and classification of CKD. Kidney Int Suppl (2011). 2013;3(1):19-62. doi:10.1038/kisup.2012.64 25018975 PMC4089693

[11] Chen J Zhang F Liu CY , et al. Impact of chronic kidney disease on outcomes after total joint arthroplasty: a meta-analysis and systematic review. Int Orthop. 2020;44(2):215-229. doi:10.1007/s00264-019-04437-4 31834442

[12] Cheng C Yan Y Zhang Q Guo W. Effect of chronic kidney disease on total knee arthroplasty outcomes: a meta-analysis of matched control studies. Arthroplasty. 2021;3(1):21. doi:10.1186/s42836-021-00078-4 35236487 PMC8796397

[13] Coresh J Selvin E Stevens LA , et al. Prevalence of chronic kidney disease in the United States. JAMA. 2007;298(17):2038-2047. doi:10.1001/jama.298.17.2038 17986697

[14] Cui A Li H Wang D Zhong J Chen Y Lu H. Global, regional prevalence, incidence and risk factors of knee osteoarthritis in population-based studies. EClinicalMedicine. 2020;29-30:100587. doi:10.1016/j.eclinm.2020.100587 34505846 PMC7704420

[15] Deegan BF Richard RD Bowen TR Perkins RM Graham JH Foltzer MA. Impact of chronic kidney disease stage on lower-extremity arthroplasty. Orthopedics. 2014;37(7):e613-e618. doi:10.3928/01477447-20140626-51 24992055

[16] Del Balso C Halai MM MacLeod MD Sanders DW Rahman Lawendy A. Factors predictive of early complications following total ankle arthroplasty. Foot Ankle Orthop. 2022;7(2):24730114221102456. doi:10.1177/24730114221102456 PMC9201329 35722173

[17] DiMagno AN Hajj-Hussein I Othmani AE Stasch J Sayeed Z El-Othmani MM. Chronic kidney disease impact on total joint arthroplasty outcomes: a national inpatient sample-based study. J Orthop Surg (Hong Kong). 2020;28(3):2309499020916129. doi:10.1177/2309499020916129 32383393

[18] Fan Z Yan L Liu H , et al. The prevalence of hip osteoarthritis: a systematic review and meta-analysis. Arthritis Res Ther. 2023;25(1):51. doi:10.1186/s13075-023-03033-7 36991481 PMC10053484

[19] Fox JA Domingue GA DeMaio CV Brockman BS Malloy K Thakral R. Total hip arthroplasty complications in patients with chronic kidney disease: a comparison study. J Orthop. 2023;39:1-6. doi:10.1016/j.jor.2023.03.013 37077839 PMC10106339

[20] GBD Chronic Kidney Disease Collaboration. Global, regional, and national burden of chronic kidney disease, 1990-2017: a systematic analysis for the Global Burden of Disease Study 2017. Lancet. 2020;395(10225):709-733. doi:10.1016/S0140-6736(20)30045-3 32061315 PMC7049905

[21] Glazebrook M Daniels T Younger A , et al. Comparison of health-related quality of life between patients with end-stage ankle and hip arthrosis. J Bone Joint Surg Am. 2008;90(3):499-505. doi:10.2106/JBJS.F.01299 18310699

[22] Jager KJ Kovesdy C Langham R Rosenberg M Jha V Zoccali C. A single number for advocacy and communication—worldwide more than 850 million individuals have kidney diseases. Kidney Int. 2019;96(5):1048-1050. doi:10.1016/j.kint.2019.07.012 31582227

[23] Jamsa P Jamsen E Huhtala H Eskelinen A Oksala N. Moderate to severe renal insufficiency is associated with high mortality after hip and knee replacement. Clin Orthop Relat Res. 2018;476(6):1284-1292. doi:10.1007/s11999.0000000000000256 29601379 PMC6263598

[24] Jamsa P Reito A Oksala N Eskelinen A Jamsen E. Does chronic kidney disease affect implant survival after primary hip and knee arthroplasty? Bone Joint J. 2021;103-B(4):689-695. doi:10.1302/0301-620X.103B4.BJJ-2020-0715.R2 33789475

[25] Karzon AL Kadakia RJ Coleman MM Bariteau JT Labib SA. The rise of total ankle arthroplasty use: a database analysis describing case volumes and incidence trends in the United States between 2009 and 2019. Foot Ankle Int. 2022;43(11):1501-1510. doi:10.1177/10711007221119148 36050924

[26] Kim CW Kim HJ Lee CR Wang L Rhee SJ. Effect of chronic kidney disease on outcomes of total joint arthroplasty: a meta-analysis. Knee Surg Relat Res. 2020;32(1):12. doi:10.1186/s43019-020-0029-8 32660587 PMC7219208

[27] Ko CJ Brooks Z Veater R Zhu S Willson KW Choung DJ. The effect of frontal deformity at the ankle joint on total ankle arthroplasty revision rate. J Foot Ankle Surg. 2024;63(2):145-150. doi:10.1053/j.jfas.2023.09.012 37805097

[28] Lee JW Im WY Song SY Choi JY Kim SJ. Analysis of early failure rate and its risk factor with 2157 total ankle replacements. Sci Rep. 2021;11(1):1901. doi:10.1038/s41598-021-81576-y 33479348 PMC7820457

[29] Levey AS Stevens LA. Estimating GFR using the CKD Epidemiology Collaboration (CKD-EPI) creatinine equation: more accurate GFR estimates, lower CKD prevalence estimates, and better risk predictions. Am J Kidney Dis. 2010;55(4):622-627. doi:10.1053/j.ajkd.2010.02.337 20338463 PMC2846308

[30] Lim S Jordan SW Jain U Kim JY. Predictors and causes of unplanned re-operations in outpatient plastic surgery: a multi-institutional analysis of 6749 patients using the 2011 NSQIP database. J Plast Surg Hand Surg. 2014;48(4):270-275. doi:10.3109/2000656X.2013.871287 24533745

[31] Martin-Fernandez J Garcia-Maroto R Bilbao A , et al. Impact of lower limb osteoarthritis on health-related quality of life: a cross-sectional study to estimate the expressed loss of utility in the Spanish population. PLoS One. 2020;15(1):e0228398. doi:10.1371/journal.pone.0228398 PMC6980637 31978194

[32] McKenna BJ Cook J Cook EA , et al. Total ankle arthroplasty survivorship: a meta-analysis. J Foot Ankle Surg. 2020;59(5):1040-1048. doi:10.1053/j.jfas.2019.10.011 32600863

[33] Miric A Inacio MC Namba RS. The effect of chronic kidney disease on total hip arthroplasty. J Arthroplasty. 2014;29(6):1225-1230. doi:10.1016/j.arth.2013.12.031 24556110

[34] Murray C Marshall M Rathod T Bowen CJ Menz HB Roddy E. Population prevalence and distribution of ankle pain and symptomatic radiographic ankle osteoarthritis in community dwelling older adults: a systematic review and cross-sectional study. PLoS One. 2018;13(4):e0193662. doi:10.1371/journal.pone.0193662 PMC5927448 29708977

[35] Paget LDA Tol JL Kerkhoffs G Reurink G . Health-related quality of life in ankle osteoarthritis: a case-control study. Cartilage. 2021;13(1 suppl):1438S-1444S. doi:10.1177/19476035211025814 PMC8808799 34165357

[36] Penner MJ Berlet GC Calvo R , et al. The demographics of total ankle replacement in the USA: a study of 21,222 cases undergoing pre- operative CT scan-based planning. Foot Ankle Orthop. 2020;5(4):2473011420S00381.

[37] Rama-Maceiras P Rey-Rilo T Moreno-Lopez E Molins-Gauna N Sanduende-Otero Y Pensado-Castineiras A. Unplanned surgical reoperations in a tertiary hospital: perioperative mortality and associated risk factors. Eur J Anaesthesiol. 2011;28(1):10-15. doi:10.1097/eja.0b013e32833e33b0 21166109

[38] Sambandam S Senthil T Serbin P Viswanathan VK Mounasamy V Wukich D. Analysis of baseline characteristics, length of stay, cost of care, complications and subgroup analysis of patients undergoing total ankle arthroplasty-a large database study. J Foot Ankle Surg. 2023;62(2):310-316. doi:10.1053/j.jfas.2022.08.007 36163143

[39] Schindelar L McEntee R D’Amore T Beredjiklian P Lutsky K. Unplanned return to the operating room in upper-extremity surgery: incidence and reason for return. J Hand Surg Am. 2021;46(8):715.e1-715.e12. doi:10.1016/j.jhsa.2021.01.019 33994259

[40] Tan TL Kheir MM Tan DD Filippone EJ Tischler EH Chen AF. Chronic kidney disease linearly predicts outcomes after elective total joint arthroplasty. J Arthroplasty. 2016;31(9 suppl):175-179.e2. doi:10.1016/j.arth.2016.03.019 27067757

[41] Theis KA Murphy LB Guglielmo D , et al. Prevalence of arthritis and arthritis-attributable activity limitation - United States, 2016-2018. MMWR Morb Mortal Wkly Rep. 2021;70(40):1401-1407. doi:10.15585/mmwr.mm7040a2 34618800 PMC8519273

[42] Venishetty N Wukich DK Beale J , et al. Total knee arthroplasty in dialysis patients: a national in-patient sample-based study of perioperative complications. Knee Surg Relat Res. 2023;35(1):22. doi:10.1186/s43019-023-00196-0 37533126 PMC10394770

[43] You Y Zhang Y Qiang L , et al. Prevalence and risk factors for perioperative complications of CKD patients undergoing elective hip surgery. J Orthop Surg Res. 2019;14(1):82. doi:10.1186/s13018-019-1118-9 30894199 PMC6425709

[44] Zaidi R Cro S Gurusamy K , et al. The outcome of total ankle replacement: a systematic review and meta-analysis. Bone Joint J. 2013;95-B(11):1500-1507. doi:10.1302/0301-620X.95B11.31633 24151270

